# Blood-brain barrier associated tight junction disruption is a hallmark feature of major psychiatric disorders

**DOI:** 10.1038/s41398-020-01054-3

**Published:** 2020-11-02

**Authors:** Chris Greene, Nicole Hanley, Matthew Campbell

**Affiliations:** 1 Smurfit Institute of Genetics, Trinity College Dublin, Dublin, 2 Ireland; 2 FutureNeuro SFI Research Centre, Royal College of Surgeons in Ireland, Dublin, Ireland

**Keywords:** Molecular neuroscience, Psychiatric disorders

## Abstract

Major psychiatric disorders affect 25% of the population. While genetic and environmental risk factors have been identified, the underlying pathophysiology of conditions, such as schizophrenia, bipolar disorder and major depression remains largely unknown. Here, we show that endothelial associated tight junction components are differentially regulated at the blood-brain barrier (BBB) in distinct neuroanatomic regions of human donor brain tissues. Previous studies have shown associations between BBB disruption and the development of psychiatric behaviours in rodents. Using immunohistochemistry and qRT-PCR, we show that the expression of claudin-5 is reduced in the hippocampus of individuals diagnosed with major depression or schizophrenia. We also show that levels of tight junction mRNA transcripts, including claudin-5, claudin-12 and ZO-1 correlate with disease duration and age of onset of a range of psychiatric disorders. Together, these data show that BBB associated tight junction disruption and dysregulation is a common pathology observed across the major psychiatric disorders. Targeting and regulating tight junction protein integrity at the BBB could, therefore, represent a novel therapeutic strategy for these conditions.

## Introduction

Mental disorders affect approximately 1 in 4 people across the world with depression, bipolar disorder and psychosis affecting >380 million people worldwide^1^. Despite the readily available treatment options, up to two-thirds of individuals will never seek help from a healthcare professional. In addition, the intolerable side-effects of many commonly prescribed anti-depressants, anti-psychotics and mood stabilizers leads to diminished usage, highlighting the necessity to identify novel biological processes perturbed in these conditions to enable the generation of new therapeutics^2^. While genetic and environmental risk factors have been associated with psychiatric disorders^3,4^, the pathophysiology still remains poorly understood.

In the central nervous system (CNS), blood vessels have distinctive properties that tightly regulate molecular exchange between the blood and brain parenchyma. This blood-brain barrier (BBB) controls the entry of vital nutrients from the circulation to the brain and the removal of waste products as well as preventing the entry of pathological agents^5^. The integrity of the BBB is essential for CNS homeostasis and damage to the capillary endothelium and BBB can contribute to the pathogenesis of various neurological disorders including epilepsy, depression and schizophrenia^6–10^. Indeed, transcriptomic studies have revealed that differentially regulated genes in schizophrenia are enriched in the vasculature^11^. Furthermore, dysfunction of the BBB, in particular the tight junction protein claudin-5, in depression, has been shown to exacerbate behavioural dysfunction in the mouse chronic social defeat stress model of depression and contribute to immune cell infiltration^6^. Similarly, loss of claudin-5 is associated with reduced acoustic pre-pulse inhibition, a schizophrenia-related behavioural abnormality^7^. Psychiatric disorders are complex multifactorial diseases that are difficult to recapitulate in mouse models, so clarification is needed regarding the association of markers of BBB integrity and psychiatric disease-related pathology in humans.

In this study, 7 regions of the human brain were analysed for tight junction proteins, which constitute the paracellular barrier at the BBB. Importantly, we compared four disease groups and assessed their associations with age of onset, duration of disease, prevalence of psychosis and other psychiatric disease-related features. Our results indicate that levels of the key tight junction protein claudin-5 are decreased in the hippocampus of human brain from individuals diagnosed with depression and schizophrenia, while mRNA and protein levels of claudin-5, claudin-12 and ZO-1 are associated with the age of onset and duration of schizophrenia, bipolar disorder and depression.

## Materials and methods

Post-mortem tissues and RNA samples were obtained through the Stanley Medical Research Foundation for psychiatric disorders. We evaluated the neuropathology consortium which is a collection of 60 brains consisting of 15 each diagnosed with schizophrenia, bipolar disorder, major depression and non-diseased control brains. Samples were collected, with informed consent from next-of-kin, by participating medical examiners between January 1995 and June 2002. Diagnoses were made by 2 senior Psychiatrists according to DSM-IV criteria. The demographics associated with each group are summarised in Table 1.

**Table 1:** Summaries of demographic, histological and clinical information for bipolar, depression and schizophrenia cases and controls.

Variable	Control	Bipolar	Depression	Schizophrenia
Age at Death, years	52 (29, 68)	48 (25, 61)	46 (30, 65)	44 (25, 62)
Sex, male, female	9,6	9,6	9,6	9,6
Age of onset, years		19 (7, 39)	32 (11, 54)	21 (13, 42)
Duration, years		21 (6, 43)	11 (1, 42)	23 (5, 45)
Post-mortem interval, hours	26 (8, 42)	28 (13, 62)	26 (7, 47)	32 (12, 61)
Relative brain mass, grams	1490 (1305, 1840)	1440 (1130, 1690)	1460 (1240, 1740)	1440 (1270, 1640)
acidity-alkalinity (log scale; 7 = neutral)	6.3 (5.8, 6.6)	6.2 (5.8, 6.5)	6.2 (5.8, 6.5)	6.2 (5.8, 6.6)
Storage time, days	286 (31, 774)	647 (224, 836)	489 (86, 931)	622 (68, 938)
Number with pyschosis	0	11	0	15
Number with suicide	0	9	7	4
Lifetime quantity of fluphenazine or equivalent, mg	0	7500 (0, 60,000)	0	35000 (0, 200,000)
Severity of alcohol abuse	1.1	2.8	1.8	1.7
Severity of substance abuse	0.1	1.9	1.1	1.2

The sample median (minimum, maximum) is given for age at death, PMI, relative brain mass, acidity-alkalinity, storage time and lifetime quantity of fluphenazine. For Severity of alcohol and severity of substance abuse, mean score is given (0—little or none, 1—social, 2—moderate use past, 3—moderate use present, 4—heavy use past, 5—heavy use present).

### Immunohistochemistry

Fresh frozen 14 µm sections from the orbital frontal cortex and hippocampus were fixed for 10 min in ice-cold methanol and subsequently rinsed briefly in PBS. Sections were blocked with 5 % normal goat serum (NGS) containing 0.05 % triton-x100 in PBS for 1 h at room temperature before overnight incubation in primary antibodies to claudin-5 (0.5 µg/ml) (ThermoFisher 34-1600), in 1% NGS. Sections were washed twice in PBS and incubated in secondary fluorescent antibodies (4 µg/ml) (Abcam ab150077) for 2 h at room temperature, before 3 washes in PBS. Nuclei were counterstained with Hoechst (1 µg/ml) for 1 min at room temperature before a final wash in PBS. Sections were cover-slipped with Aqua Poly/mount and left to air-dry in the dark before imaging. Images were taken on a Zeiss LSM 710 confocal microscope. Images were analysed in ImageJ by thresholding the images and calculating the integrated density. 5–10 images were analysed per patient from the grey and white matter. Additionally, vessel density and vessel diameter were measured. For immunohistochemistry, 2 samples were depleted from the hippocampus cohort.

### qRT-PCR

Transcript levels were quantified using a two-step protocol on the StepOne Plus^TM^ Real-Time PCR System (Applied Biosystems). cDNA was synthesised from 500 ng of RNA isolated from the premotor frontal cortex (Broadmann Area (BA6), parietal cortex (BA7), caudal cingulate cortex (BA23/31), occipital cortex (BA18/19) and cerebellum, using a High-Capacity cDNA Reverse Transcription Kit (Applied Biosystems). Real-time PCR was performed using the Sensifast SYBR Hi-ROX Kit (Bioline) according to the manufacturer’s instructions. RT-PCR conditions were as follows: 95 °C × 2 min; (95 °C × 5 s; 60 °C × 30 s) × 40; 95 °C × 15 s, 60 °C × 1 min, 95 °C × 15 s, 60 °C × 15 s. Relative gene expression levels were measured using the comparative *C*_T_ method (ΔΔ*C*_T_). Expression levels of target genes were normalized to the housekeeping gene *ACTB*. Primer sequences for human *CLDN5*, *CLDN12*, *OCLN*, *TJP1*, *TJP2*, *PECAM1*, and *ACTB* are listed in the table below.

**Table:** 

**Target**	**Forward**	**Reverse**
*CLDN5*	CTGGACCACAACATCGTGA	CACCGAGTCGTACACTTTGC
*CLDN12*	AGTAAAATGCCCTGCGTGTG	AGGCAAGCTTGTGGAGTACT
*OCLN*	TCAGGGAATATCCACCTATCACTTCAG	CATCAGCAGCAGCCATGTACTCTTCAC
*TJP1*	CGGTCCTCTGAGCCTGTAAG	GGATCTACATGCGACGACAA
*TJP2*	ATCAGACTAGCCACTCCTGC	AACCCAGTCCCACAAACAGA
*PECAM1*	TCAGGCAACGCACAAAACAG	GACCTGCTCGGTTCTCTCTG
*ACTB*	GGGAAATCGTGCGTGACAT	CAGCGGAACCGCTCATTGCCAATGG

### Statistical analysis

All analysis was performed blind to diagnosis with clinical details revealed only when full quantitative datasets were provided to the Stanley Medical Research Foundation. Multiple groups analysis was performed with a Kruskal–Wallis test with a Dunn’s post-test to correct for multiple comparisons. A *P* value < 0.05 was considered statistically significant. For correlation analysis (*n* = 24 correlations), Spearman’s rank order correlation was performed with the strength of the correlations considered as follows: *r* < 0.3 weak; 0.3 < *r* < 0.6 moderate; *r* > 0.6 strong with multiple comparisons corrected using the Benjamini, Krieger and Yekutieli method with a False Discovery Rate (FDR) of <0.25. For correlation analysis, we tested the association between the age of onset and duration of illness with levels of claudin-5 protein or mRNA as assessed by immunohistochemistry and qRT-PCR respectively.

## Results

### Characteristics of non-diseased, bipolar, depression and schizophrenia disease patient groups

Table 1 details the characteristics of each of the four disease groups. There were no significant differences between age at death (*P* = 0.62) and post-mortem interval (PMI) (P = 0.21) between the groups. There was a significantly increased severity of substance abuse (*P = 0.0159) and alcohol abuse (*P = 0.0363) in bipolar patients.

### Microvessel densities and claudin-5 levels differ between grey and white matter

**Fig. 1:**
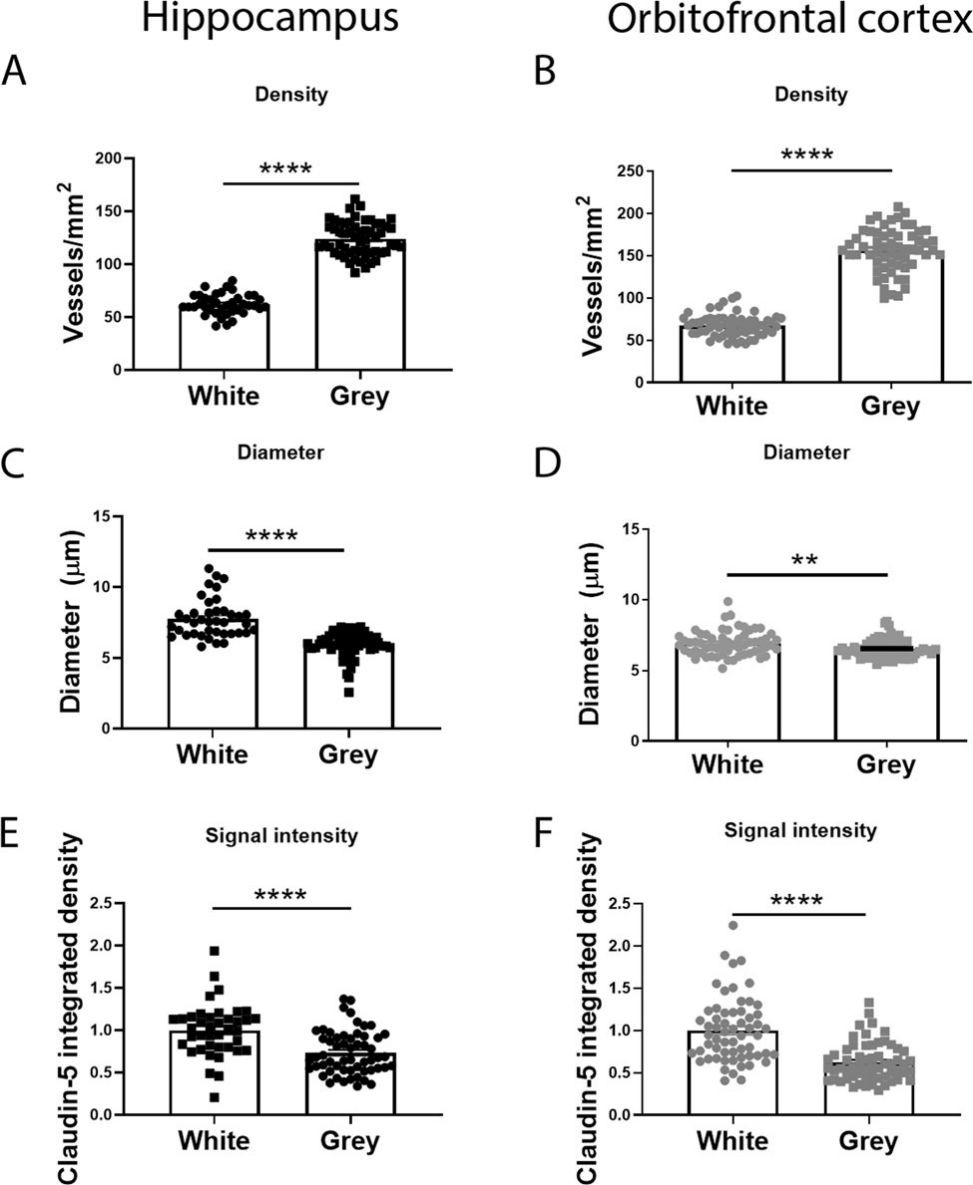
Comparison between grey and white matter vessels in the orbitofrontal cortex. **A**, **B** Vessel density quantified as number of vessels per mm^2^ in the white and grey matter in the hippocampus and orbitofrontal cortex. **C**, **D** Diameter of vessels (µm) in the white and grey matter in the hippocampus and orbitofrontal cortex. **E**, **F** Claudin-5 signal intensity in the white and grey matter in the hippocampus and orbitofrontal cortex. Data represent means ± SEM; each symbol is one patient. Mann–Whitney test for comparisons. **P < 0.01, ****P < 0.0001.

**Fig. 2:**
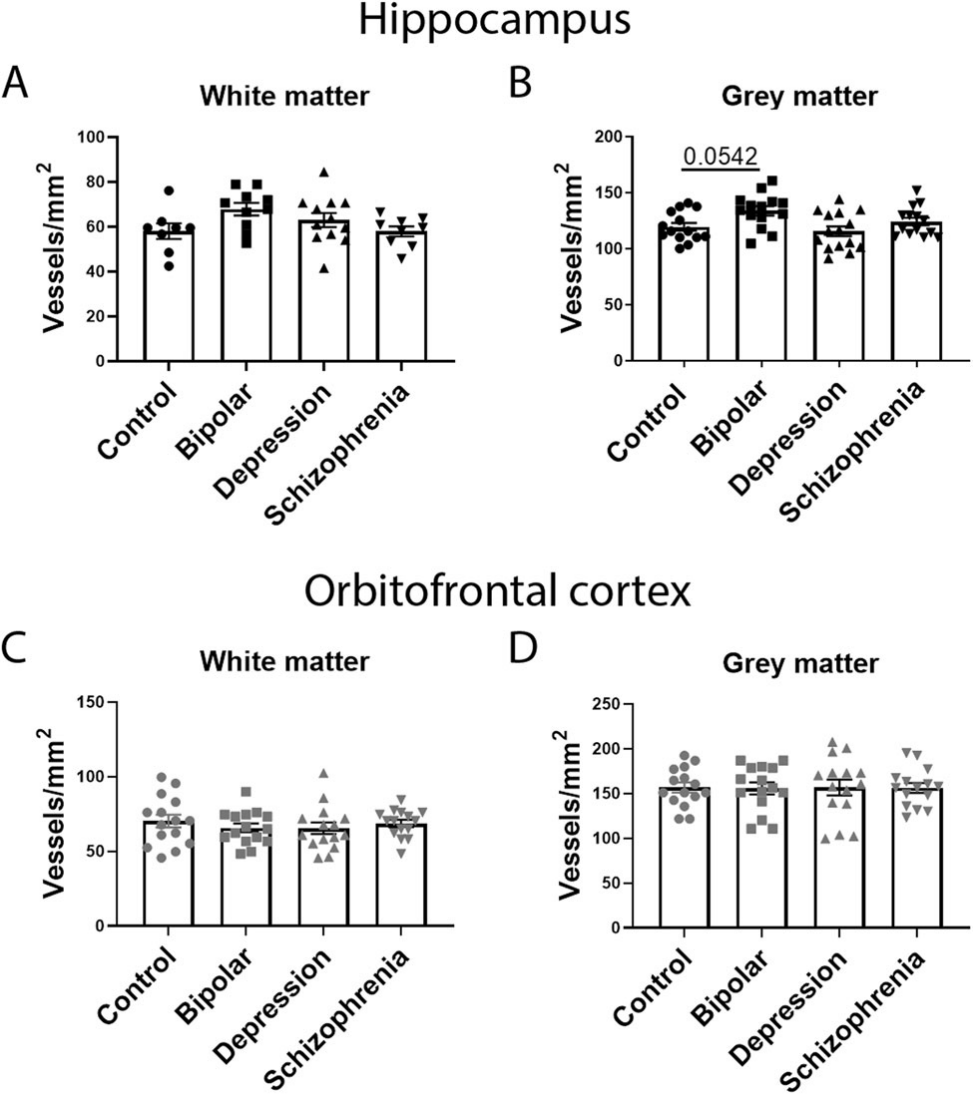
Vessel density compared between all disease groups. **A** Vessel density in the hippocampus white matter. **B** Vessel density in the hippocampus grey matter. **C** Vessel density in the orbitofrontal cortex white matter. **D** Vessel density in the orbitofrontal cortex grey matter. Data represent means ± SEM; each symbol is one patient. Kruskal–Wallis one-way ANOVA followed by Dunn’s post-test. *P < 0.05.

**Fig. 3:**
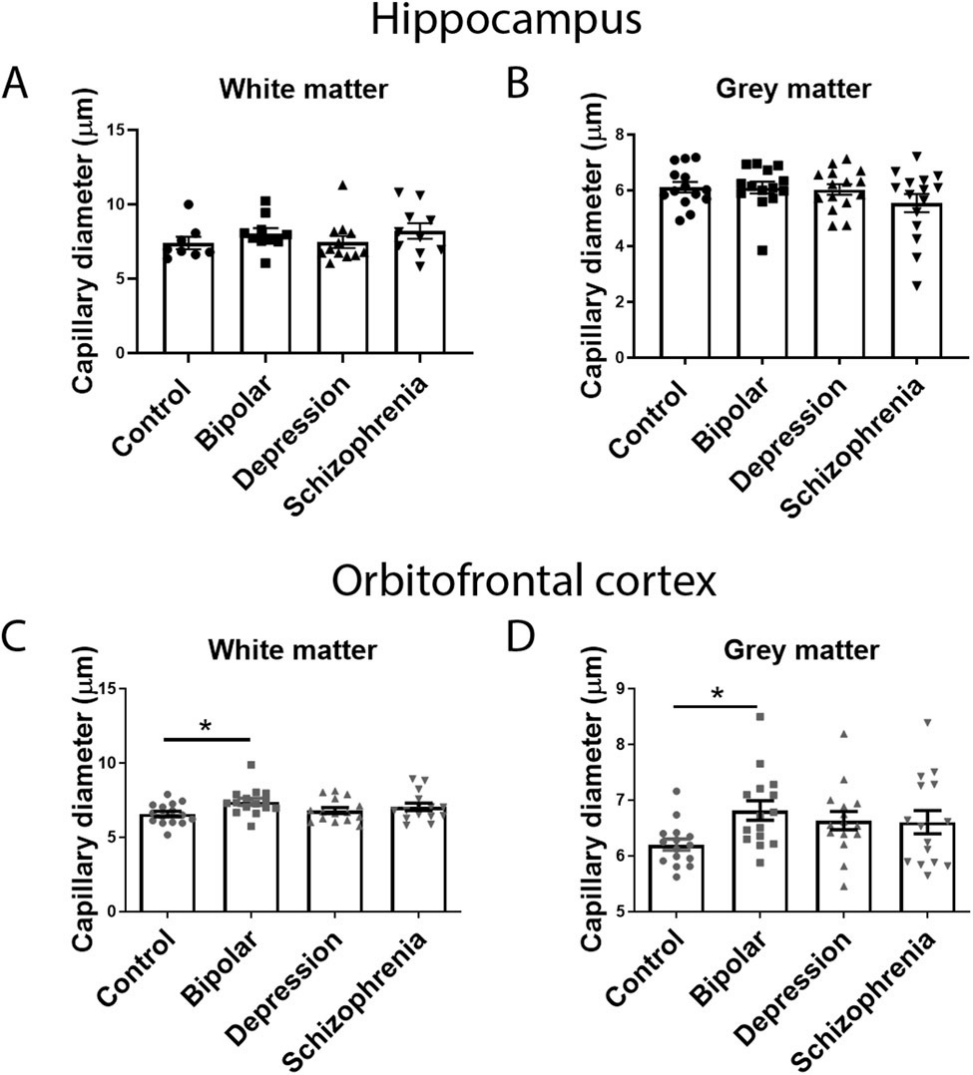
Vessel diameter compared between all disease groups. **A** Vessel diameter in the hippocampus white matter. **B** Vessel diameter in the hippocampus grey matter. **C** Vessel diameter in the orbitofrontal cortex white matter. **D** Vessel diameter in the orbitofrontal cortex grey matter. Data represent means ± SEM; each symbol is one patient. Kruskal–Wallis one-way ANOVA followed by Dunn’s post-test. *P < 0.05.

First, we found increased vessel density (****P < 0.0001), reduced vessel diameter (****P < 0.0001) and reduced claudin-5 staining intensity (****P < 0.0001) in the grey matter versus the white matter in the hippocampus (Fig. 1A, C, E) and orbitofrontal cortex (Fig. 1B, D, F). Therefore, tight junction histological analysis was performed on grey and white matter separately. There was a non-significant increase in vessel density in the grey matter hippocampus of the bipolar patient group (*P* = 0.054) (Fig. 2B) while there was increased vessel diameter in the bipolar patient group in the grey (*P = 0.023) and white matter (*P = 0.0342) of the orbitofrontal cortex (Fig. 3C, D).

### Tight junction proteins are decreased in the hippocampal area of patients with depression and schizophrenia

**Fig. 4:**
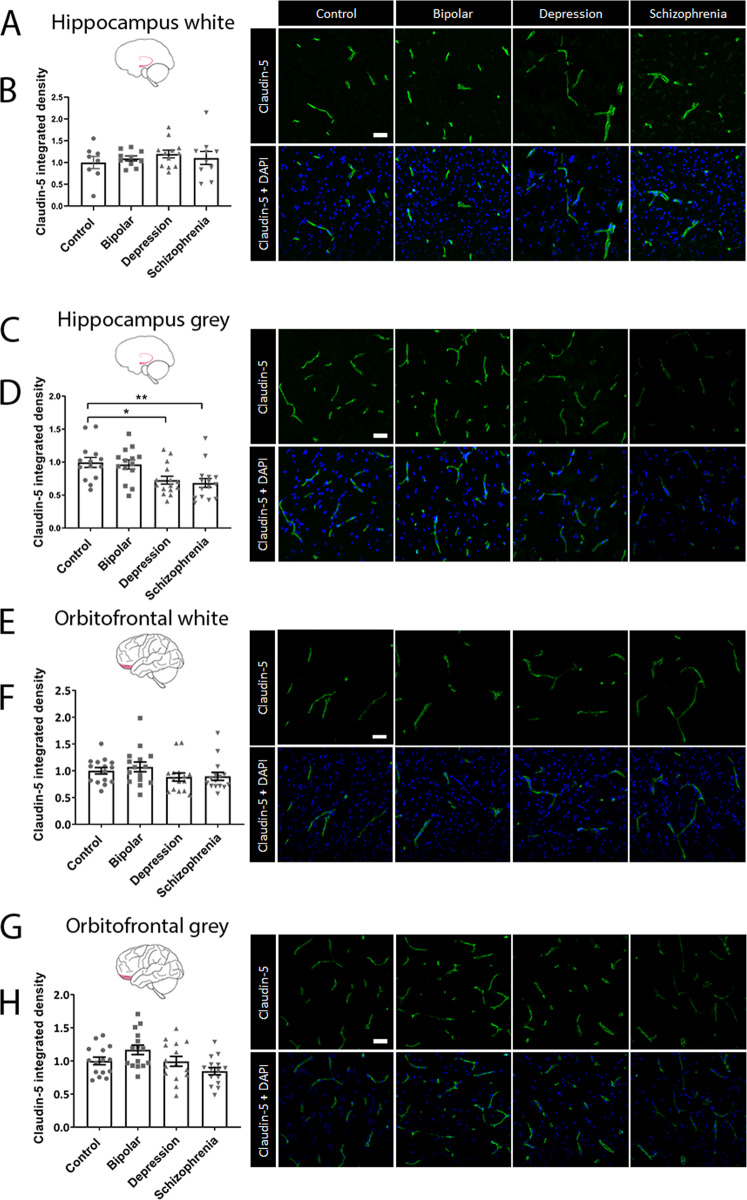
Immunohistochemical analysis of claudin-5 protein in the hippocampus and orbitofrontal cortex. **A**, **C**, **E**, **G** Representative images of claudin-5 staining in each of the four disease groups for each brain region. Scale bar = 50 µm. **B**, **D**, **F**, **H** Integrated density of claudin-5 in the hippocampus white matter, hippocampus grey matter, orbitofrontal cortex white matter and orbitofrontal cortex grey matter respectively. Data represent means ± SEM; each symbol is one patient. Kruskal–Wallis one-way ANOVA followed by Dunn’s post-test. *P < 0.05, **P < 0.01.

To determine whether the expression of tight junction proteins is altered in any of the psychiatric disorders, the levels of the most enriched tight junction protein, claudin-5, were quantified by immunohistochemistry in the orbitofrontal cortex and hippocampus of each of the four disease groups. We observed significantly reduced claudin-5 levels in the depression (*P = 0.0158) and schizophrenia (**P = 0.0073) patient groups in the hippocampus grey matter (Fig. 4C, D). No differences were observed in any other patient group or brain region (Fig. 4A, B, E–H).

### Levels of claudin-5 are correlated with age of onset and duration of psychiatric disorders

**Fig. 5:**
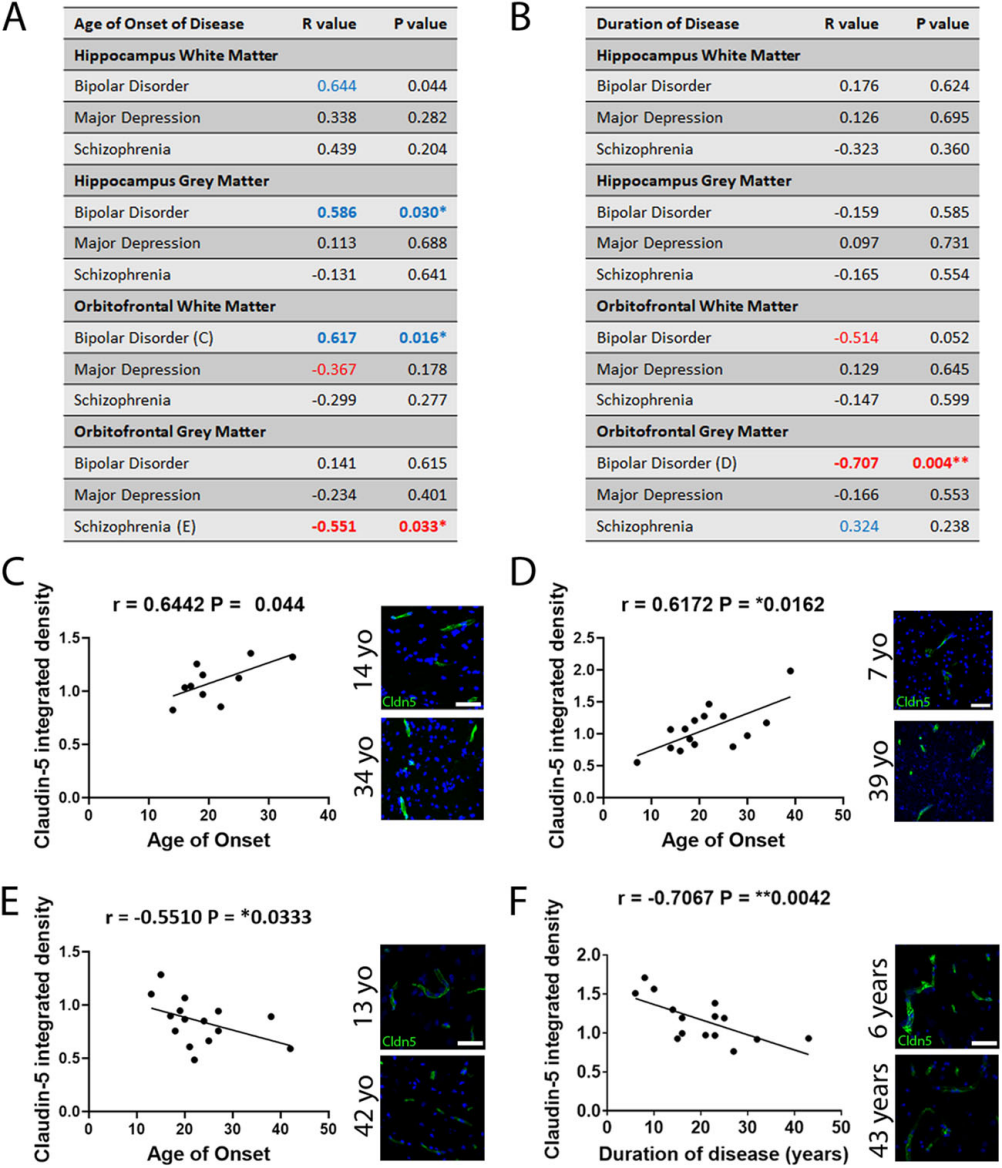
Correlation between claudin-5 protein levels and age of onset of or duration of each disease in the hippocampus grey matter and orbitofrontal cortex white and grey matter. **A**, **B** Spearman *r* values and *P* values for correlations between age of onset (**A**) and duration (**B**) of disease. Strong correlations highlighted (*r* > 0.3 and *r* < −0.3). *P < 0.05, **P < 0.01. **C** Scatter plot of claudin-5 signal vs age of onset of bipolar disease in hippocampus white matter. Representative images of claudin-5 in an individual with bipolar disorder at an age of onset of 14 and 34 years old. **D** Scatter plot of claudin-5 signal vs age of onset of bipolar disease in orbitofrontal cortex white matter. Representative images of claudin-5 in an individual with bipolar disorder at an age of onset of 7 and 39 years old. **E** Scatter plot of claudin-5 signal vs age of onset of schizophrenia in orbitofrontal cortex grey matter. Representative images of claudin-5 in an individual with schizophrenia at an age of onset of 13 and 42 years old. **F** Scatter plot of claudin-5 signal vs duration of bipolar disease in orbitofrontal cortex white matter. Representative images of claudin-5 in an individual with bipolar disorder for 6 years and 43 years. Yo, years old.

We next performed correlation analysis to identify any associations between levels of claudin-5 and the age of onset (Fig. 5A) or duration (Fig. 5B) of psychiatric illness in each patient group and brain region. Levels of claudin-5 positively correlated with the age of onset of bipolar disorder in the hippocampus white matter (*r* = 0.6442, *P* = 0.044 (not significant with FDR correction)) (Fig. 5A, C), grey matter (*r* = 0.586, *P = 0.030) (Fig. 5A) and orbitofrontal white matter (*r* = 0.6172, *P = 0.0162) (Fig. 5A, D). Claudin-5 levels negatively correlated with the age of onset of schizophrenia in the orbitofrontal grey matter (*r* = −0.5510, *P = 0.0333) (Fig. 5A, E). Levels of claudin-5 negatively correlated with the duration of bipolar disorder in the orbitofrontal white matter (*r* = −0.5135, *P* = 0.0522) and orbitofrontal grey matter (*r* = −0.7067, **P = 0.0042) (Fig. 5B, F).

### Claudin-5 protein levels do not associate with common features of psychiatric disorders

We next investigated associations between some of the common features of psychiatric disorders with levels of claudin-5 protein; including incidence of psychosis and suicide. Spearman’s rank order correlations assessed relationships between claudin-5 levels and other parameters provided in patient demographic information including age at death, brain pH, post-mortem interval, relative brain mass, storage time, lifetime quantity of antipsychotics taken (given as fluphenazine equivalents) and gender. No significant correlations were observed for any parameter (Supplementary Fig. 1A, C, E, G). There were no differences in the levels of claudin-5 in patients with psychosis in any disease group and similarly no association was found between drug and alcohol abuse or use of anti-psychotics and claudin-5 protein levels (Supp Fig. 1). Levels of claudin-5 were significantly increased in the orbitofrontal grey matter of individuals who died by suicide (*P = 0.0267) (Supplementary Fig. 1H).

### Tight junction mRNA levels associate with features of psychiatric disorders

**Fig. 6:**
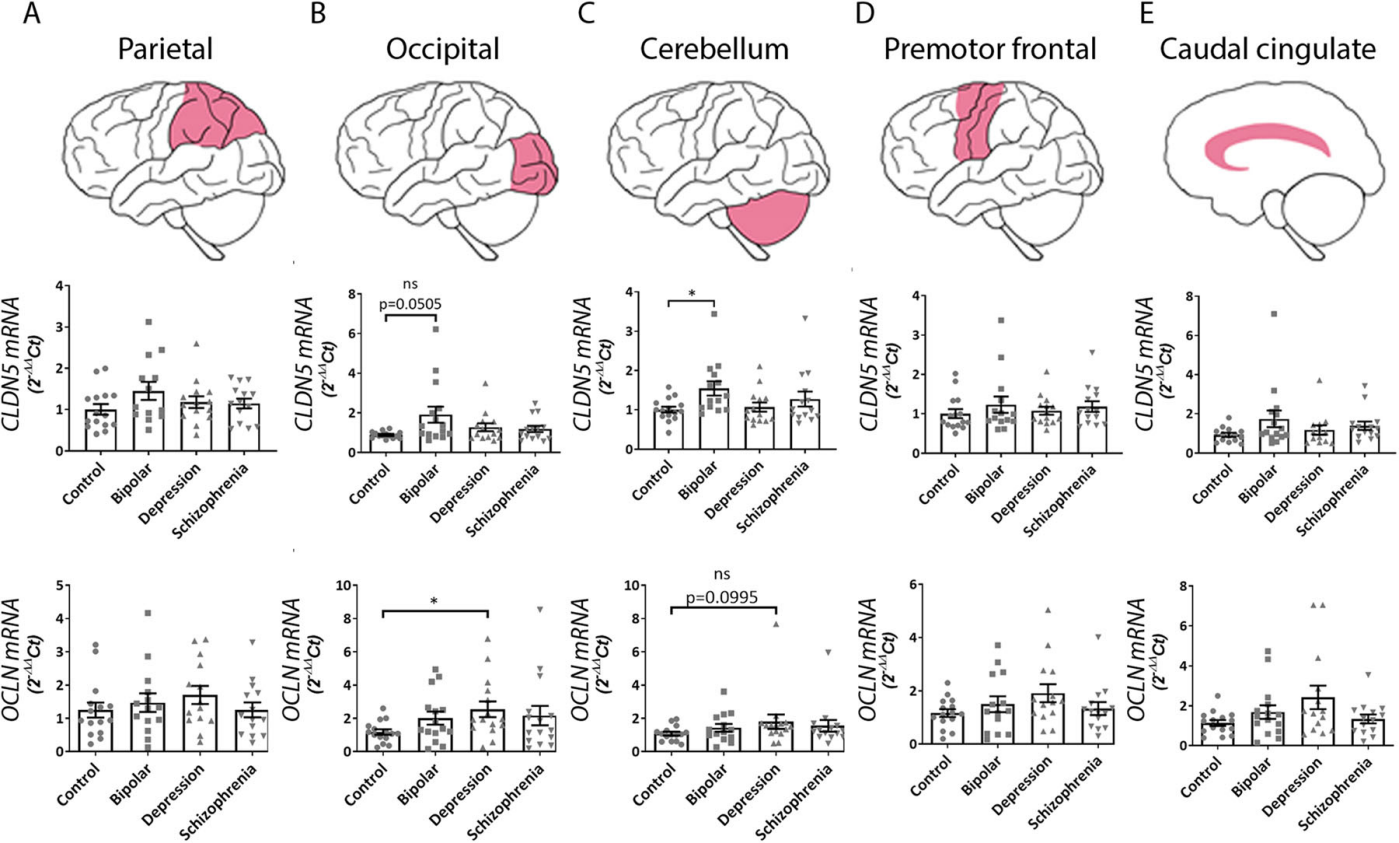
mRNA expression levels of tight junction proteins compared between diseases in five brain regions. qPCR analysis of *CLDN5* and *OCLN* mRNA expression levels in **A** parietal cortex, **B** occipital cortex, **C** cerebellum, **D** premotor frontal cortex and **E** caudal cingulate cortex. Graphs represent 2^−ΔΔCt^ values normalised to *ACTB*. Unless otherwise stated results are non-significant. Data represent means ± SEM; each symbol is one patient. Kruskal–Wallis one-way ANOVA followed by Dunn’s post-test. *P < 0.05, ns, not significant.

We next investigated the mRNA expression levels of claudin-5 and occludin in the parietal cortex, occipital cortex, cerebellum, premotor frontal cortex, and caudal cingulate cortex across the four disease groups. *CLDN5* mRNA was increased in the occipital cortex (*P* = 0.0505) and cerebellum (*P = 0.0284) in bipolar brains (Fig. 6B, C), while *OCLN* was significantly increased in the occipital cortex (*P = 0.0397) of depression brains (Fig. 6B). We also investigated mRNA levels of *CLDN12*, *TJP1*, *TJP2* and *PECAM1* with no significant differences observed (Supplementary Fig. 2).

### Tight junction mRNA levels associate with age of onset and duration of psychiatric disorders

**Fig. 7:**
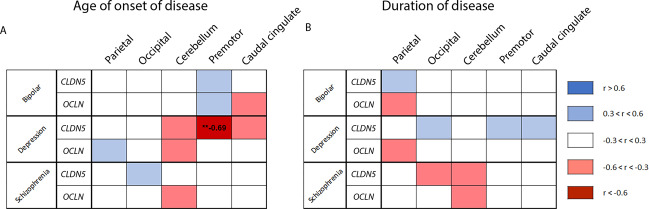
Correlation matrix for age of onset and duration of disease. Correlation matrix between tight junction mRNA expression levels and age of onset (**A**) or duration (**B**) of bipolar disorder, major depression and schizophrenia. **A** spearman *r* values for the correlation between *CLDN5* or *OCLN* mRNA expression and age of onset of disease across each disease group. **B** spearman r values for the correlation between *CLDN5* or *OCLN* mRNA expression and duration of disease across each disease group. Strength of correlations is colour coded according to the legend. Significant *r* values included. **P < 0.01.

Correlation analysis revealed several moderate and strong correlations between mRNA levels of tight junction components and age of onset and duration of disease across each of the disease groups. Levels of *CLDN5* (*r* = −0.694, *P < 0.01) and *CLDN12* (*r* = −0.578, *P < 0.05) were negatively correlated with the age of onset of depression in the premotor frontal (Fig. 7A) and cerebellum (Supplementary Fig. 3A) respectively while levels of *CLDN12* (*r* = −0.69, *P < 0.01) were negatively correlated with age of onset of schizophrenia in the caudal cingulate cortex (Supplementary Fig. 3A). Additionally, levels of *TJP1* (*r* = −0.561, *P < 0.05) and *CLDN12* (*r* = −0.56, *P < 0.05) were negatively correlated with the duration of bipolar disorder in the occipital cortex and premotor frontal cortex respectively while *CLDN12* (*r* = −0.547, *P < 0.05) was negatively correlated with the duration of schizophrenia in the cerebellum (Supplementary Fig. 3B).

## Discussion

Overall, our results suggest that the expression of junctional components is altered across a spectrum of psychiatric disorders with the most pronounced effects observed in patients diagnosed with depression and schizophrenia. We focussed on the major tight junction protein claudin-5. Claudin-5 maintains the integrity of the paracellular aqueous channel of adjacent brain endothelial cells and strictly regulates the movement of small molecules, ions as well as peripheral cytokines. Knockout of claudin-5 in mice results in post-natal lethality with size-selective loosening of the BBB to molecules up to 1 kDa in size^12^. Furthermore, loss of claudin-5 in the nucleus accumbens is associated with depressive phenotypes while loss of claudin-5 in the hippocampus and medial prefrontal cortex is associated with schizophrenia-related behaviours in rodents^6,7^. We found reduced expression of claudin-5 in the hippocampus of patients diagnosed with depression and schizophrenia. This is in line with previous studies which found claudin-5 deficits in the nucleus accumbens of patients diagnosed with major depression^6^. Previous work has also identified loss of claudin-5 in the prefrontal cortex of schizophrenia patients; however, we found no differences in claudin-5 levels in the orbitofrontal cortex of our schizophrenia cohort. It is possible that claudin-5 disruption in the prefrontal cortex is highly region specific^13^. We have also previously reported on the discontinuity of claudin-5 immunoreactivity in the parietal lobe of schizophrenia patients which we further linked to the presence of the rs10314 variant in the *CLDN5* gene.

Analysis of tight junction transcripts revealed discordant results with significant increases in *CLDN5*, *OCLN* and mRNA in bipolar and depression brains samples. Correlation analysis revealed that there was a consistent and robust negative correlation between the expression of tight junction mRNAs and the duration of psychiatric disease with longer duration of disease associated with reduced expression of *CLDN12* and *TJP1* notably. ZO-1 acts as a scaffolding protein to tether junctional proteins including claudin-5 and occludin to the actin cytoskeleton^14^. Recently, however, the role of claudin-12 at the BBB has been investigated with studies in knockout mice demonstrating it is not involved in maintenance of BBB integrity^15^. Whereas claudin-5 is specifically expressed in endothelial cells in the central nervous system, it must be noted that occludin, ZO-1 and ZO-2 are also expressed in other CNS cell types. Therefore, the apparent lack of change in these mRNAs may not accurately reflect changes in endothelial levels of the respective mRNAs^16^.

It is increasingly recognised that dysfunction of the BBB has a causative role in the pathogenesis of psychiatric disorders^8,17^ in models of human psychiatric disorders while in humans, clinical studies have identified consistent increases of the astrocytic protein S100β in the serum of patients diagnosed with schizophrenia. S100β is typically confined to the brain parenchyma and its presence in serum correlates with BBB disruption. Increased concentrations of serum S100β are also associated with psychotic features of schizophrenia and depressive features in major depression disorder^18–22^. While studies assessing BBB disruption via levels of serum S100β are becoming more common, it must be noted that S100β may not reflect BBB disruption via disruption of tight junction components. Indeed, changes to the rate of cerebrospinal fluid (CSF) flow and drainage may impact S100β levels in the blood while increased production by astrocytes and subsequent drainage into blood via CSF may also impact measurements and not simply reflect BBB breakdown. Similar issues have also been raised for measurements of the albumin CSF:serum ratio^17^.

More recent evidence has revealed regulatory mechanisms in brain endothelial cells that can prevent the onset of psychiatric disorders. In the chronic social stress defeat model of depression, low endothelial levels of FoxO1 and HDAC1 are associated with stress resilience^23^. FoxO1 is a known repressor of claudin-5 expression^24^. In another study, depleted levels of neuronal cAMP in the nucleus accumbens was associated with stress susceptibility and reduced BBB integrity^25^. This suggests that targeting and regulating proteins involved in BBB function could be a potential therapeutic strategy for psychiatric disorders. Imaging of BBB function is also a potential tool in the diagnosis and management of psychiatric disorders. In a subgroup of bipolar disorder patients, dynamic contrast-enhanced MRI (DCE-MRI) revealed extensive BBB dysfunction that was associated with greater psychiatric morbidity^26^. Developing a greater understanding of the contribution of BBB disruption and dysfunction in psychiatric disorders may lead to the development of the next generation of therapeutic modalities for these devastating conditions.

### Limitations

As with all studies concerning post-mortem human tissue samples, there are some limitations of the approaches used. Firstly, while we focused our study on BBB components, the nature of the collection of the samples resulted in a heterogenous mixture of cell types. Therefore, it is difficult to interpret the results of some components due to their expression in several cell types in the brain. As such, we may miss out on discrete differences in expression of proteins or mRNAs in a BBB-specific context. Another limitation to post-mortem studies are the several confounding factors such as lifetime drug use. As we did not have access to specific types of medications used by patients in the consortium, it is difficult to elucidate the relative contribution of drug use to expression of junctional components. While we could look at the association of overall quantities of medication, we could not determine the effect of duration or specific subsets of medication on levels of BBB components. Another confounding factor is the length of time between death and post-mortem examination, however in our study there were no significant differences between PMI and each of the disease groups or controls.

BBB disruption has been associated with cognitive function, for example in the context of dementia in Alzheimer’s Disease where individuals with early cognitive dysfunction develop damage to the brain capillary network and BBB breakdown in the hippocampus irrespective to changes in amyloid β or tau biomarkers. This suggests that BBB breakdown is an early biomarker of human cognitive dysfunction^27^. While we did not have clinical measurement of cognitive function in our study, we did find consistent associations between the age of onset of illness and the levels of claudin-5 with earlier onset of bipolar disorder associated with reduced levels of claudin-5. We also found that reduced levels of claudin-5 were associated with increased duration of bipolar disorder. This may suggest that cumulative disease burden results in more severe BBB breakdown. Other studies have found age-dependent breakdown of the BBB in the hippocampus of individuals with mild cognitive impairment although future studies are warranted to investigate if this is the case in psychiatric patients^28^.

## Supplementary information

Supplementary Figure 1

Supplementary Figure 2

Supplementary Figure 3

Supplementary Figure Legents
