# Supplementary figures and images for: Blood-brain barrier associated tight junction disruption is a hallmark feature of major psychiatric disorders
Source: Transl Psychiatry. 2020 Nov 2;10:373. doi: 10.1038/s41398-020-01054-3 (PMC7606459; doi:10.1038/s41398-020-01054-3)

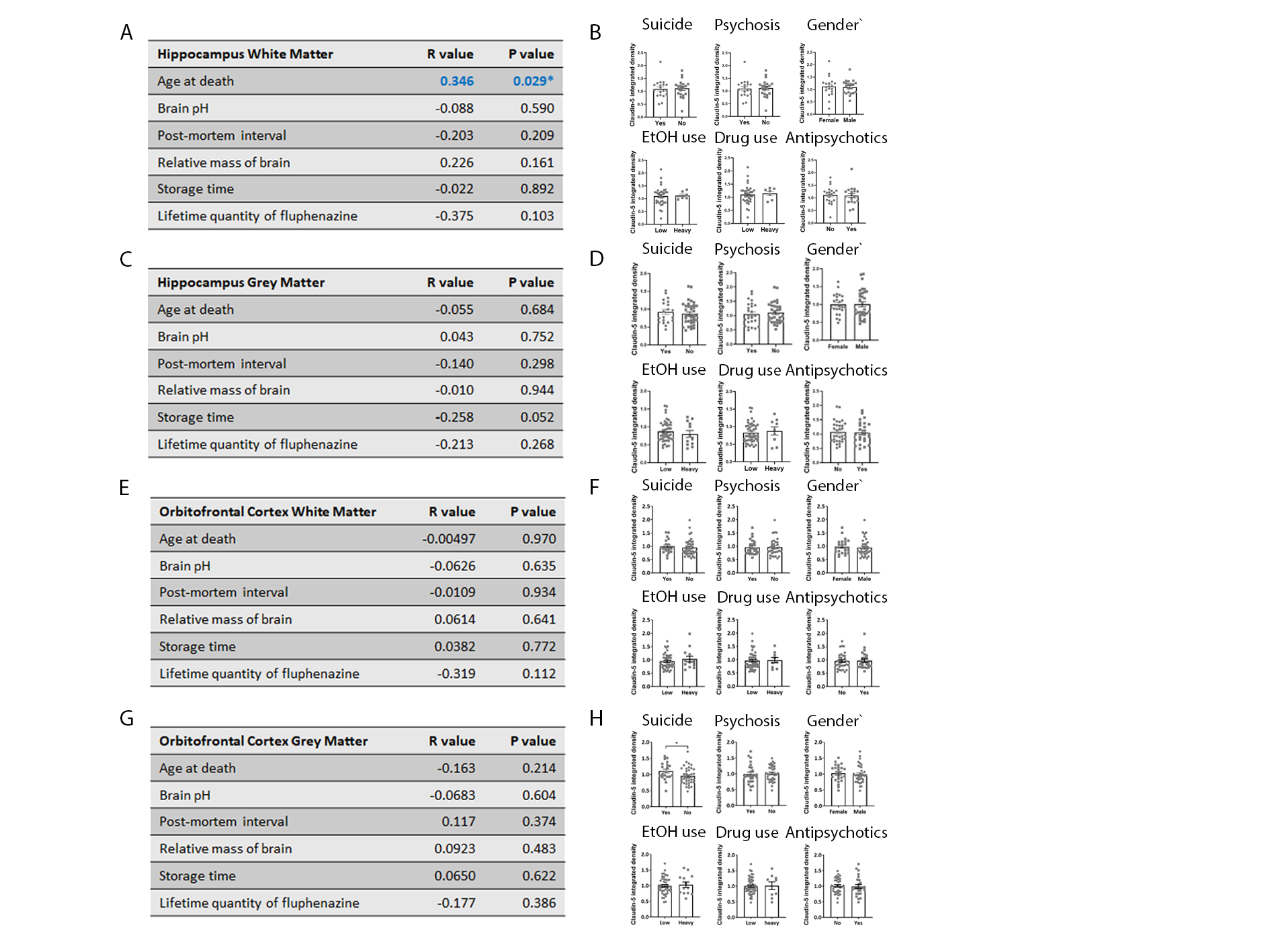

Supplement: Supplementary file 1 — Supplementary Figure 1 [file 41398_2020_1054_MOESM1_ESM.tif]

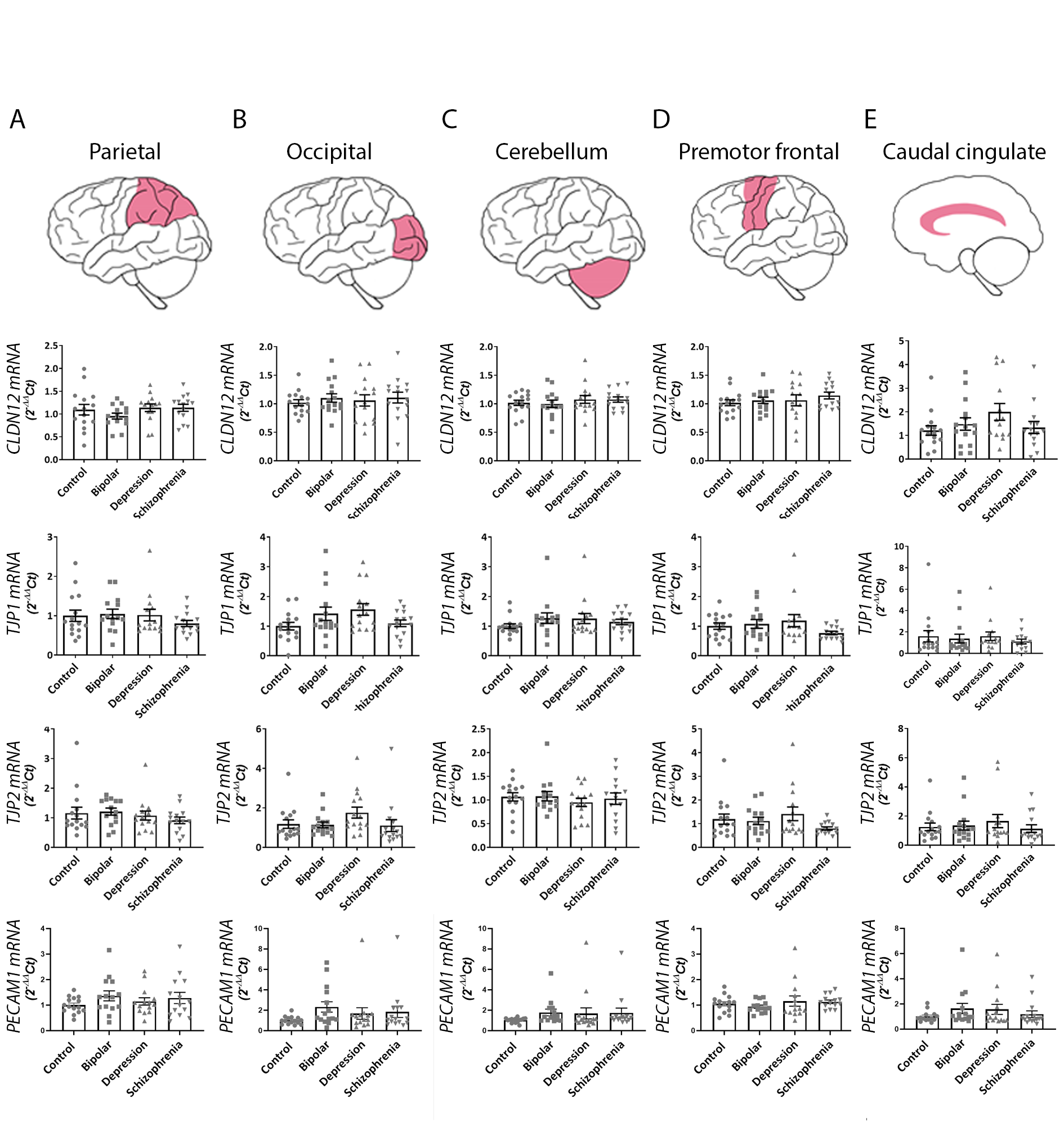

Supplement: Supplementary file 2 — Supplementary Figure 2 [file 41398_2020_1054_MOESM2_ESM.tif]

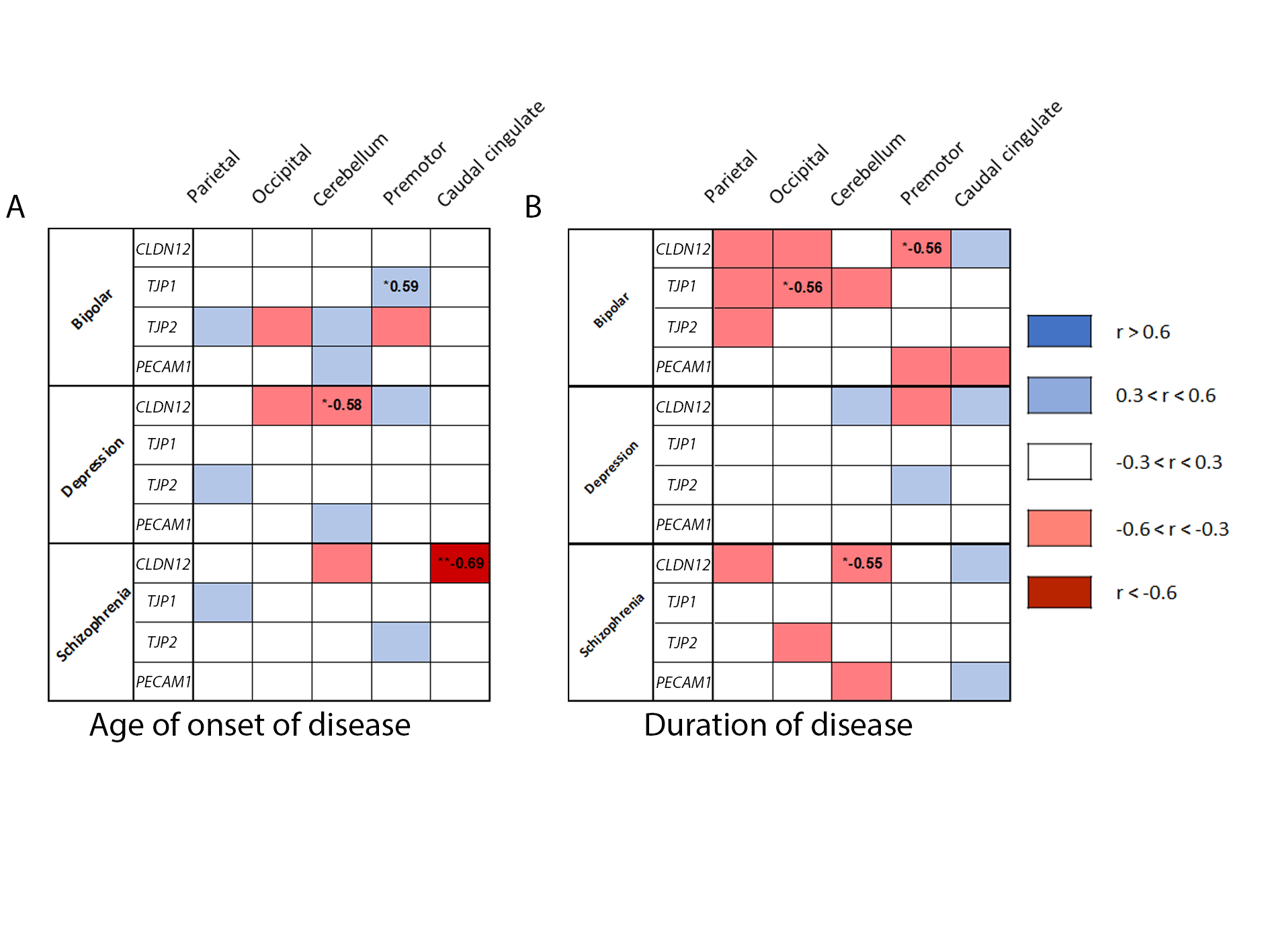

Supplement: Supplementary file 3 — Supplementary Figure 3 [file 41398_2020_1054_MOESM3_ESM.tif]
