# Supplementary material for: Blood-brain barrier associated tight junction disruption is a hallmark feature of major psychiatric disorders
Source: Transl Psychiatry. 2020 Nov 2;10:373. doi: 10.1038/s41398-020-01054-3 (PMC7606459; doi:10.1038/s41398-020-01054-3)
Supplement: Supplementary file 4 — Supplementary Figure Legents [file 41398_2020_1054_MOESM4_ESM.docx]

**Supplementary Figure 1. Effect of demographical, histological and clinical parameters on claudin-5 protein levels. (**A, C, E, G) Spearman r values and P values for correlations between claudin-5 signal intensity and age at death, brain pH, post-mortem interval, relative mass of brain, storage time and lifetime quantity of anti-psychotics as fluphenazine equivalents in each brain region. (B, D, F, H) Effect of suicide, psychosis, gender and use of alcohol, drugs or antipsychotics on claudin-5 levels in each brain region. *P < 0.05.

**Supplementary Figure 2. mRNA expression levels of tight junction proteins compared between diseases in five brain regions.** qPCR analysis of *CLDN12*, *TJP1*, *TJP2* and *PECAM1* mRNA expression levels in (A) parietal cortex, (B) occipital cortex, (C) cerebellum, (D) premotor frontal cortex and (E) caudal cingulate cortex. Graphs represent 2^-ΔΔCt^ values normalised to *ACTB*. Data represent means ± SEM; each symbol is one patient. Unless otherwise stated results are non-significant.

**Supplementary Figure 3.** **Correlation matrix between tight junction mRNA expression levels and age of onset (A) or duration (B) of bipolar disorder, major depression and schizophrenia.** A Spearman r values for the correlation between *CLDN12*, *TJP1*, *TJP2* or *PECAM1* mRNA expression and age of onset of disease across each disease group. B spearman r values for the correlation between *CLDN12*, *TJP1*, *TJP2* or *PECAM1* mRNA expression and duration of disease across each disease group. Strength of correlations is colour coded according to the legend. Significant r values included. *P < 0.05, **P < 0.01.
